# What does being “research active” mean in public health practice? Exploring behavioural and motivational dimensions of research activity in English local government through perceptions from embedded researchers

**DOI:** 10.1007/s10389-024-02369-x

**Published:** 2025-01-02

**Authors:** Rachael C. Edwards, Dylan Kneale, Claire Stansfield, Sarah Lester

**Affiliations:** Evidence for Policy and Practice Information Centre, UCL Social Research Institute, Institute of Education, University College London, Gower Street, London, WC1E 6BT UK

**Keywords:** Evidence-based practice, Local government, Knowledge transfer, Research system

## Abstract

**Aim:**

Developing policies and services to address health inequalities occurs within highly complex, political environments, and the literature points to an underutilisation of research. This ineffective mobilisation of evidence is a result of challenges emerging from both research and practice organisations. In response, many interventions have been funded to enhance “research activity” in public health decision-making, but we lack a holistic understanding of what characterises research activity in these settings. Addressing this gap, we explored behavioural and motivational dimensions that are viewed as comprising research activity in public health practice.

**Subject and methods:**

We undertook seven semi-structured interviews with researchers embedded in local government settings in England. As change agents holding dual affiliations with research and practice organisations, embedded researchers are ideally placed to develop comparative insights on the processes through which research activity is manifested in these contexts and to identify disparities inhibiting the flow of research evidence into practice.

**Results:**

We found that research activity is manifested through a variety of behaviours relating to accessing, conducting, and contributing to research, and evidence use. Motivational dimensions underlying engagement with research institutions include concerns around capability, capacity, and local value. Many of these concerns stem partly from narrow conceptualisations of the term “research” which are held by academics and decision-makers alike.

**Conclusion:**

Interventions seeking to enhance research activity in public health decision-making could benefit from flexibility in their language and application to build cross-organisational understanding of pressures and priorities and to account for variation in research capacity, interest, and skillsets.

## Introduction

A large body of literature points to an underutilisation of research in the formation of public health policies and services (Fafard 2015; Kneale et al. 2017). This lack of evidence-informed decision-making can lead to ineffectual public spending and reduce the potential for public health decisions to address health inequalities (Fynn et al. 2021). A growing body of work has explored the barriers underlying research inactivity within public health (e.g., Orton et al. 2011; van der Graaf et al. 2021), identifying many constraints within policy/practice organisations such as capacity limitations, insufficient research infrastructure, and the political nature of decision-making (Coates and Mickan 2020; Edwards et al. 2024a; Homer et al. 2022; Kneale et al. 2019). Research institutions equally contribute to inhibiting the translation of research into practice, for example, by undertaking research that is misaligned with public health priorities and through poor engagement with practice organisations (Kneale et al. 2017; van der Graaf et al. 2017; Williams et al. 2020). Ultimately, reducing the research–practice divide will require cultural changes and greater collaboration across research institutions and public health organisations.

Funding bodies are increasingly acknowledging the need to address barriers hindering the translation of research into practice within public health. This recognition is evidenced through the funding of innovative partnerships between practice settings and research organisations (e.g., embedded researchers) and additional support to improve research capacity and capability. For example, the National Institute for Health and Care Research (2022) Health Determinants Research Collaboration fund in England recently invested £50 million in research–policy partnerships “*to embed a culture of evidence-based decision-making within local government*”. In another example, the Canadian Institutes of Health Research Health System Impact Fellowship programme supported impact-oriented postdoctoral career paths, aiming to build research capacity within health system organisations (Kasaai et al. 2023). Aligning with this funding investment, researchers exploring evidence use within public health commonly identify the aim of improving research activity. For example, Homer et al.’s (2022) research on local governments situated in the north of England “*explored the assets that exist to foster a stronger research culture*”.

While healthcare bodies are increasingly extolling the benefits of organisations becoming more “research active” (e.g., Australian Academy of Health and Medical Sciences 2022; National Institute for Health and Care Research 2024; NHS 2023), the way in which “research activity” is understood is open to interpretation. For example, a research-active culture could be framed as a complex blend of behaviours (e.g., service evaluation, participation in broader research ecosystems) that interventions aim to enhance by addressing underlying challenges such as capacity and capability constraints. This complexity is often poorly articulated by organisations calling for the need to enhance research activity in practice settings, leading to ambiguity regarding the specific behaviours and challenges interventions should seek to influence and the processes through which they can activate this change. We contend that this lack of clarity results in part from poor theoretical grounding. While some logic models have begun to emerge in the context of embedded researchers (Cheetham et al. 2019; Kneale et al. 2023), there is a need to continue developing our theoretical understanding of research cultures and the interventions aiming to influence them.

### Behaviours reflecting a research-active culture

A large body of work has investigated the complex and political process of evidence use in public health decision-making (Kneale et al. 2019; Liverani et al. 2013; Orton et al. 2011). Although other aspects of research activity have also been explored, such as through van der Graaf et al.’s (2021) work investigating the concept of co-production and associated mechanisms within an English local government context, little research has focused explicitly on unpacking the behaviours that reflect a research-active culture in public health practice. One notable exception is Fynn et al. (2021), who explored research activity in Norfolk County Council, England, broadly defining it as “*activities inclusive of conducting research and using evidence from research*”. Through a diverse mix of qualitative methods, they found that common examples of research activity included “*ongoing use of evidence in service improvement and development plans; public consultations; drawing on evidence from other local authorities informally and formally; and devising tools, methods and interventions, testing implementation, and evaluation*”. Expanding on this work, we seek to further elucidate dimensions of research activity within local government public health settings to provide more concrete grounding for interventions seeking to improve cultures of research.

### Motivation to engage in research activity

In addition to identifying desired behavioural outcomes, interventions aiming to enhance research activity will need to incorporate motivational processes within their theories of change. More specifically, such theories will need to account for the values, beliefs, and perceptions among public health practitioners that impact their engagement with research as well as the interrelationships between these factors and wider determinants of research activity (e.g., senior support, research funding) (Edwards et al. 2024b). Some motivational aspects of research activity have been explored in the context of public health. For example, in their interview-based research with public health professionals in England, van der Graaf et al. (2017) found that participants “*recognised the importance of research findings for informing their practice*”. Other studies have investigated how different forms of evidence are valued within public health decision-making, often finding that local evidence is prioritised (Homer et al. 2022; Kneale et al. 2019). More work is needed to unpack the complexity underlying motivation to engage with research, including the relationships between these dimensions and broader institutional barriers and opportunities.

### Aim and research questions

We aim to provide an in-depth understanding of the behavioural and motivational dimensions comprising research activity within public health settings in the UK. In England, local government—referred to as local authorities (LAs)—public health teams have directed the delivery of most municipal public health services and set the direction for local public health policy since 2013 (Kneale et al. 2017). LAs thus present ideal settings in which to develop our understanding of research activity in the context of addressing health inequalities. Through perceptions of embedded researchers in English LA public health teams, this research addressed two questions in the context of public health practice:

1. What behaviours reflect dimensions of research activity?
2. What values, beliefs, and perceptions inhibit and facilitate research activity?

The purpose of this research is not to provide a definitive assessment of the extent to which certain behaviours and motivational dimensions are present, but rather to identify the breadth of these concepts within public health.

Embedded researchers are a novel type of intervention to improve the translation of evidence into practice (Coates and Mickan 2020). These roles are co-located in research and policy/practice settings, have continual engagement with both organisations, and are directed by the broad aim of enabling research production and use (Barratt et al. 2017; Kneale et al. 2024; McGinity and Salokangas 2014). As a result of their position as institutional bridges and change agents, these roles can address many of the aforementioned barriers to evidence use (Cheetham et al. 2018; Reen et al. 2022). They are also ideally placed to develop unique, contextualised insight into the processes through which research activity is manifested within their host organisation. As such, they can offer a more holistic, alternative perspective to traditional understandings of research activity which tend to be framed through the lens of academia.

## Methods

### Approach: perceptions from embedded researchers

We explored behavioural and motivation dimensions of research activity through semi-structured interviews with researchers embedded within diverse LA public health teams in England. The embedded researcher posts of interest in this study were referred to as Public Health Local Authority Research Practitioners (PHLARPs) and were funded by the National Institute for Health and Care Research (NIHR) as part of its Clinical Research Network (CRN). In addition to being affiliated with one or more LA public health team, PHLARPs were linked to a research organisation (either a university or the CRN). The overarching aim of the PHLARP programme was to enhance cultures of research within LA public health settings. Our research team has investigated the PHLARP programme from a variety of perspectives, with the present research reporting on one component of this broader study (Kneale et al. 2023).

Most of the PHLARPs were culturally embedded within LA teams, participating in day-to-day activities and establishing close working relationships with LA colleagues. While becoming embedded within their LA, many PHLARPs carried out a situation analysis to gain an understanding of the local research context and identify the primary barriers and opportunities influencing levels of research engagement (Edwards et al. 2024b). Based on this knowledge, they undertook a wide range of research-enabling activities (e.g., establishing connections between the LA and research institutions, supporting research projects, research production). Through these efforts, PHLARPs gained a contextually bound understanding of the ways in which behavioural and motivational dimensions of research activity was manifested within their affiliated LA. For example, they could provide an understanding of the research behaviours that are desirable within LAs, but also feasible. Given the diversity that existed across PHLARPs, they also presented an opportunity to explore variation in research activity across public health settings. We drew together elements commonly expressed by PHLARPs and examined points of variation.

### Recruitment and interview protocol

The CRN provided contact details for 24 PHLARPs from their programme, all of whom were invited to participate. We conducted two rounds of interviews with PHLARPs, with the present study reporting on data from the seven interviews that formed round 2 (March–April 2023). At this point, PHLARPs had been in post for 2 to 2.5 years. The interview schedule was piloted with one PHLARP, but as no substantial changes were warranted, they were included in the sample. All interviews were conducted online through Zoom. PHLARPs were informed of the anonymity of their responses and provided written consent to participate. This research was approved by a University College London Institute of Education research ethics committee (REC1540). All interviews were audio-recorded, lasting an average of 59 min (range: 51–65 min), and manually transcribed.

The initial portion of the interview focused on exploring dimensions of research activity within LA public health teams. We asked PHLARPs what they perceived to constitute a research-active culture from their experience working within the LA. PHLARPs were asked to focus on the behaviours they had observed, or were working towards promoting, relating to research. We then asked PHLARPs to reflect on the changes they had observed in these behaviours during their time in post.

We then explored motivations among LA staff for engaging in research. We asked PHLARPs to discuss the values, beliefs, and perceptions among their LA colleagues that they perceived to affect engagement with research. PHLARPs were then asked to reflect upon changes they had observed in these motivational dimensions. Throughout our interviews, we prompted PHLARPs to provide specific, illustrative examples.

As a fellow researcher, the interviewer was a professional peer to the PHLARPs. However, she was also an outsider to the PHLARP’s experience of research within public health practice. This positionality added unique value to this study. For example, PHLARPs regularly compared their experience of research within the LA to university research systems, which we suggest was partially motivated by perceptions that the interviewer’s understanding of research was contextualised within academia.

### Overview of participants

Most of our participants (*n* = 6) worked full time in their PHLARP roles, while one split their time across this role and their position at a university. Most (*n* = 6) were positioned within a single layer of local government (e.g., city council, London borough). The final participant worked particularly closely with one LA but was also tasked with influencing local governments across a region. Although most of our participants had some level of engagement with senior LA staff (e.g., councillors), most worked more closely with staff internal to their public health team, which often included the Director of Public Health.

Six participants described themselves as being strongly embedded within their LA public health team. For four of these participants, the strength of this embeddedness was greater than they experienced with their research institution. The final participant indicated that their embeddedness within the LA was somewhat weak, but that they worked more closely with particular members of staff and community groups.

### Analysis

We analysed interviews through an inductive thematic approach (Braun and Clarke 2006) using NVivo (released in March 2020). During the transcription phase, a preliminary list of codes relating to our two research questions was identified. Codes were then added to this list and grouped into themes through an iterative coding process. All transcripts were reviewed twice for coding accuracy. This thematic analysis was undertaken by the first author, with the second author double-coding all transcripts using the first author’s thematic framework. Following detailed discussion, the authors agreed that the overarching framework accurately captured themes represented in the data. Any disagreements on the codes assigned to the text were discussed between the first and second author and edits made accordingly. Overall, there was a strong level of agreement, and only a small minority of excerpts were assigned new codes.

## Results

### Research-active behaviours among public health decision-makers

PHLARPs identified a wide range of behaviours that they perceived as dimensions of research activity within LAs. We grouped these behaviours into five broad themes: (i) types of research undertaken in LAs, (ii) research governance, (iii) accessing research, (iv) research partnerships, and (v) applying research. Across PHLARP responses, greater emphasis was placed on behaviours related to research production than to accessing or applying research evidence.

#### Types of research undertaken in public health practice

Service evaluation was identified as a dominant type of research production within LAs. For example, a PHLARP discussed how “*most of the research that you could possibly do in the local authority, at least from what I’ve observed, has to do with evaluation of existing interventions*”. LAs want to understand the health impacts of their services, how they can be improved, if they are cost-effective, and whether they should be recommissioned. For example, when reflecting on the change in research activity they had observed, a PHLARP noted that “*people are now starting to want to evaluate work that’s being done and finding out if it’s going to be cost-effective, or if it’s going to be worth ruling out*”.

LAs also regularly undertake public consultations (e.g., through surveys and focus groups), eliciting the views and priorities of residents to inform strategy development such as through Joint Strategic Needs Assessments. In the case of both public consultation and evaluation, PHLARPs often aided their LA in improving the rigour of this evidence production through, for example, advising on data collection methods. A PHLARP described such a process: “*They run surveys across their service users regularly. What’s happening differently now is they’re coming to me before they send the surveys out. And they say, ‘Can you tell me if this looks okay?’*”

#### Research governance

PHLARPs contrasted LA-based research to that led by research institutions, with the latter often referred to as “academic”, “formal”, or “proper” research. A PHLARP described this variation: “*Although people in local authorities do research, they don’t do similar research to what universities do, and there’s a whole different system, approaches, divisions, services*.” This distinction was predominantly based on universities having ethics and peer review publication processes that were often lacking within LAs.

PHLARPs described the added time needed for LAs to link into a universities’ ethics system should they wish to seek ethical approval, a challenge considering existing capacity constraints and time-sensitive research needs. Furthermore, because research governance systems akin to academia were not in place, PHLARPs discussed how LA research did not tend to be published within peer-reviewed journals, but also that such publication was often not a priority for LAs: “*As soon as it comes to, you need ethical permissions to make it an academic piece, that’s where it drops because for [the LA’s] work they don’t need it because they are not sharing that data. […] Publications are a currency for me, but not for them*.” In some cases, however, LAs possessed internal ethics processes which varied in scale and formality. For example, one of the PHLARPs established an internal research advisory group to review all incoming planned research.

#### Research partnership arrangements

Partnerships with research organisations were regularly discussed, reflecting another dimension of research activity. The extent to which LAs participate in research production as part of these collaborations varies substantially. In some cases, LAs commission research organisations to undertake a piece of work, with little ongoing involvement. For example, a PHLARP noted, “*If [the LA] wanted a service to be evaluated, they would hire a private consultancy or something to do the work. They would outsource the actual work. So, they are not doing it, but they have thought about it*.” PHLARPs also provided several examples in which their LA contributed to academic research through, for example, advising on research priorities, providing light-touch feedback on research projects, connecting academics into the community (e.g., to facilitate public involvement), and promoting research opportunities across the local area and within the LA.

Greater levels of collaboration also take place between LAs and research organisations, including co-design in which LAs provide insight and shape the project throughout its lifespan. For example, when speaking about a recent piece of research undertaken in partnership with a university, a PHLARP described how “*we co-designed the whole intervention with them, so it was a collaborative effort*”. A few of the PHLARPs discussed how such collaboration can improve the relevance of research to the LA and foster greater local support. For example, a PHLARP identified the benefits of public health co-design throughout the research process: “*The passion is there right across the board, and you can just tell the difference when you’ve got people in a team working together for something rather than just providing support*.”

Another type of partnership in which LAs are frequently involved is community engagement. This dimension of research activity was described as going beyond gathering local priorities to actively involving residents and community groups within research, as well as service and policy development. As one PHLARP described, “*We also wanted to co-create the proposal with [local stakeholders]. We wanted to have patient and public involvement from the beginning. So, we created a community steering group*.” Benefits that were identified in relation to community engagement included strengthening trust with the local community, enhancing the relevance of research, and accessing research that was already being conducted by local charities and community organisations.

#### Accessing research

Public health teams access existing evidence through a wide range of channels. The extent to which they consult peer-reviewed literature varies substantially across LAs. In some cases, established links with academic institutions and/or internal research support staff meant LA staff could access literature reviews and other research articles with relative ease and did so on a regular basis. For example, a PHLARP noted, “*If [public health staff] are interested in a topic, they could just ask the librarian to do the search for them and come up with the brief. And we have a public health intelligence and analyst team, and if anyone has any question whatsoever about anything, [that team] could find you the data on it. And then give you a report as well*.” However, not only was this support lacking in many LAs, but systems often did not even allow access to the literature. Several PHLARPs discussed the significant time and effort they had invested in establishing this access for colleagues.

Accessing existing evidence also regularly took place through a variety of alternative channels such as journal clubs and through more accessible research outputs (e.g., videos, blogs). PHLARPs also described how their public health colleagues engaged in cross-LA learning to, for example, consult about new interventions with which other LAs had experience. For example, a PHLARP indicated that in the context of addressing an emerging public health issue, “*The first thing we do is not looking up literature, it’s to consult [a nearby] Council because they are bigger, and they probably do [similar] projects*.” Although publishing in peer-reviewed journals is not often a priority for LAs, their internal evidence can still often be accessed through verbal consultation or, for example, within Joint Strategic Needs Assessment reports.

#### Evidence use

PHLARPs described several examples in which local evidence had been applied in the context of service delivery and policy development, primarily focused on evidence emerging from evaluations and public consultations. Little was said, however, on how existing academic research is used to shape public health decisions. One PHLARP emphasised the value of recognising the stages within policy cycles which present key opportunities for academics to influence LA decisions through their research: “*There would be specific points in time where research and evidence can be plugged in in the most effective way to help shape and understand and inform what local authorities are trying to do*.”

### Motivation to engage in research activity

We identified six psychological dimensions feeding into LA staff’s motivation to engage with research: (i) perceived feasibility of undertaking research, (ii) perceived value of research, (iii) attitudes towards academia, (iv) interest in research, (v) conceptualisations of research, and (vi) confidence in research abilities. Many of these dimensions relate to institutional barriers constraining research activity such as budgetary constraints and a misalignment between LA and academic priorities.

#### Perceived feasibility

All PHLARPs emphasised the severe capacity constraints experienced by their LA colleagues and perceptions among colleagues that conducting research was unfeasible. Research does not tend to be incorporated within most LA public health practitioner job descriptions, and “I don’t have time” was identified as a common initial reaction to research opportunities, with other activities such as service delivery prioritised. For example, a PHLARP described how research is “*almost seen as a thing that’s a bit of an annoyance, at the moment, because it interferes with the current work*”. As such, PHLARPs discussed the need to make their colleagues aware of the resources that were available to support research activity (including themselves) as well as the diversity of ways in which colleagues could become involved in research, many of which were minimally resource-intensive. For example, a PHLARP described how “*when we initially started [working on the funding application], the first thing was that people need to be convinced that this is something we could put in, that it’s feasible*”.

#### Perceived value

Motivation to engage with research was also influenced by the perceived value of the research to residents. While most PHLARPs had observed their LA colleagues to recognise the value of evidence-informed decision-making, the immediate value of academic-led research to the community was not always apparent, limiting involvement. As such, PHLARPs emphasised the importance of clearly articulating this value and aligning research with the strategic priorities of the LA: “*It’s not enough just to just to present things that are clearly fantastic […]. [Research] needs to be very much tailored to the local context. It needs to be informed by specific local needs that you’re then demonstrably meeting*.” PHLARPs reflected on instances where co-design enhanced the immediate value of research and thus fostered local buy-in.

#### Attitudes towards academia

Attitudes towards academia within the LA are also likely to influence LA staff’s willingness to engage with research. Unfortunately, academics are often viewed with a certain degree of wariness due to, for example, negative prior experiences in which the LA received little benefit from academic research involvement. Many PHLARPs described being on the receiving end of such negativity themselves because of their association with a research organisation. Examples of initial reactions PHLARPs received from LA colleagues included “*Why is someone from the university contacting me to try to pester me about research and stuff?*”, “*Who is the NIHR to tell us what to do and what not to do?*”, and “*Ahh, you’re one of them swotty types*”. To overcome such perceptions, PHLARPs described the need to build trust with colleagues and distance themselves from academic institutions. For example, one PHLARP described how they would introduce themselves as being based in a local city rather than a university “*because that almost sets the person off as ‘they’re an academic’. Even little, small things that could really help*”. Similarly, another PHLARP described removing academic links within email signatures.

#### Interest in research

PHLARPs also discussed how colleagues possessed naturally varying levels of interest in research. Unsurprisingly, LA staff with a research background were more likely to proactively demonstrate enthusiasm for research involvement. For example, a PHLARP described how “*there were people who were excited, and they got in touch with me immediately and said, ‘I want to do more research, how can I do it?*’”. PHLARPs spoke about identifying those most interested in research who could become champions and promote research across their teams. These PHLARPs would tailor their communications about research opportunities, requesting different levels of involvement based on initial levels of interest.

#### Conceptualisations of research

PHLARPs also spoke about how fostering a shared understanding of the word “research” can be critical to encouraging local involvement. For example, LA staff often did not perceive local evaluation as research:

*Initially [the LA staff] thought that whatever they were doing, “Oh, it’s not research, it’s just an evaluation […]”. I said, the only difference between what you are doing and research, whatever you think goes on in the university, is that there is no research ethics and governance. That’s all. Everything else, the processes you are following, are pretty much the same*.

Similarly, PHLARPs described how such internally produced evidence was often not considered as research by academics: “*Councils are doing research in terms of they are doing data collection, and they are interpreting the results, and those results are influencing policy decisions or practice decisions. But a university or any academic setting would not consider that as ‘research’ in quotes because it’s not peer-reviewed, maybe the robustness of the methodology would be questioned*.” As such, PHLARPs sought to broaden and establish a shared understanding of “research” within LAs and across stakeholders.

#### Confidence in research abilities

Finally, a few PHLARPs spoke about apprehension to engage with research among colleagues due to a lack of confidence: “*There’s almost that kind of uncertainty of taking that first step into research*.” However, the presence and knowledge of the PHLARPs themselves was perceived as reassuring to staff, helping to ease these initial hesitancies. Research training organised by the PHLARPs further addressed such apprehension.

## Discussion

**Fig. 1:**
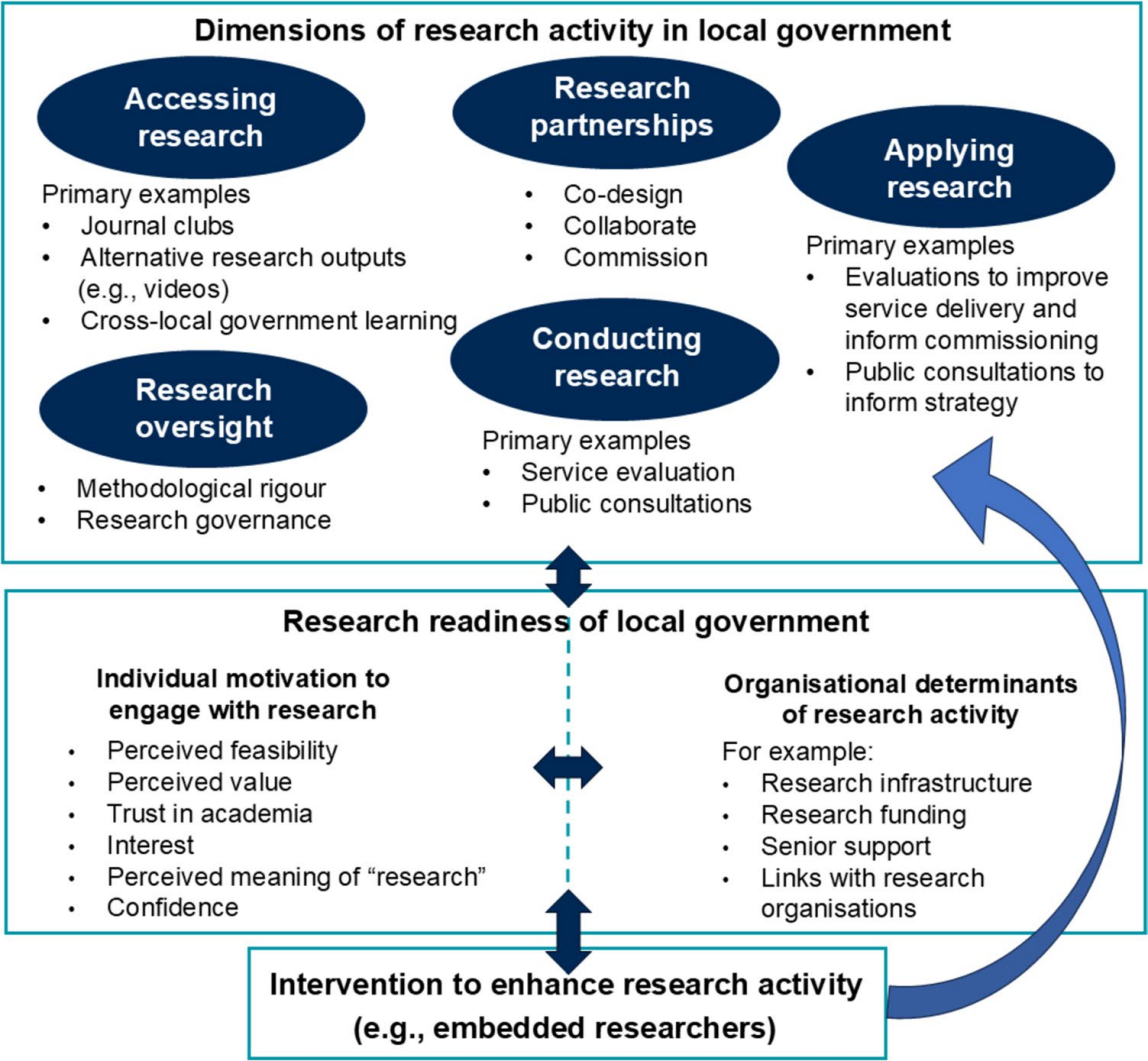
Framework conceptualising dimensions of research readiness and activity within local government and their interrelationships. Also depicted are the relationships between these factors and interventions aiming to influence research activity

We found public health research activity within LAs to be a multidimensional construct comprising the numerous behaviours involved with generating, accessing, and applying research (Fig. 1). Motivation to engage with research is similarly complex in these contexts and strongly linked to wider barriers and opportunities associated with the local government’s organisational structures and processes. Considering the identified connections across motivational and behaviours dimensions of research engagement, the following sections draw together findings from our two research questions into three dominant aspects of research engagement in public health and discuss associated implications.

### Comparing and contrasting evidence production in academia and local government

Many public health teams regularly evaluate their services and gather data on their residents’ priorities in-house and/or commission this work to non-academic research consultancies. However, in many cases, aspects of this evidence production are approached differently than research in academic settings. Although some LAs have their own research governance procedures, much of their locally produced evidence is not subject to ethical review. Due to this lack of governance, along with other factors such as concerns around confidentiality, local evidence is not often transparently published. As a result, the potential valuable contribution of such work to the broader body of knowledge is not fully realised. Research collaborations can link LAs to university ethics systems, but this process can be highly time-consuming (van der Graaf et al. 2017). Considering the resource pressures and time-sensitive research needs of LAs, the benefits of publication often do not justify such resource expenditure. Therefore, we suggest that universities should seek to simplify their ethics systems and contractual processes to accommodate feasibility constraints and work with LAs to build and enhance in-house research governance systems.

Despite these distinctions between academic and LA-based research, there are also overlaps. Public health professionals often possess research degrees (Homer et al. 2022), and several of our interviewees suggested that the research methods applied by their colleagues in local evidence production were akin to academia in many respects. However, due in part to the lack of governance processes, evidence production within LAs tends not to be perceived as “research” by academics or the LA themselves. On the contrary, for LA staff, the word “research” can elicit concerns about their capability and capacity. Fynn et al. (2021) similarly found that activities such as service evaluations were perceived as “business as usual” rather than research. Such beliefs have implications for mechanisms of fostering relationships between academia and local government, underscoring the importance of language clarity.

In their qualitative research exploring a funding scheme that linked researchers to public health practitioners in England, van der Graaf et al. (2017) identified examples in which researchers appeared to hold prejudicial views about the rigour of LA-based evidence production. Such assumptions and a failure to recognise the value of locally produced evidence can contribute to a sense that academics are out of touch with the practicalities of public health decision-making. A failure to acknowledge the overlaps in research production in these settings also presents a missed opportunity to combat perceptions about the infeasibility of research engagement. Regardless of the level of academic involvement, interventions seeking to improve research activity in local government could, where necessary, contribute to pragmatically strengthening the rigour of locally produced evidence and support the incorporation of evaluations into service design.

### Tailored research involvement can maximise uptake of opportunities

LA involvement with academic research is highly varied and adaptable. Co-design with decision-makers has many benefits, including strengthening the local relevance of research (van der Graaf et al. 2021), but there are numerous other types of collaborative approaches than can be adopted across research organisations and local government. Our results align with Fynn et al. (2021), who framed research collaboration as a continuum “*moving from engagement of external research partners in a consultative relationship or providing access to data, services or participants for externally led research at one end, to co-produced jointly led or internally led research projects […] at the other*”. Collaborative approaches across this spectrum can add significant value to academic research. Where co-design is not deemed appropriate or feasible, LAs can, for example, provide lighter-touch feedback at strategic points in the research process, promote research opportunities across the organisation, and link academics into the community. When approaching LAs, academics should seek to understand the LA’s desired level of involvement.

Levels of involvement in research can be equally varied across a public health team based on individual factors including capacity, skills, and interests. Tailoring communication with individuals based on these traits can enhance research engagement while reducing resource waste. Undertaking such targeted communication requires in-depth knowledge about LA staff and systems.

### Value and uptake of research to public health decisions

Our findings suggest that academics can enhance LA engagement by aligning research with strategic priorities and clearly articulating its local value. These results align with the large body of work highlighting the value decision-makers assign to locally produced evidence (Homer et al. 2022; Kneale et al. 2019; Li et al. 2015). Indeed, examples of evidence use identified in this study most often related to local data. In particular, our findings concur with previous work suggesting that evaluations are a primary focus for evidence production within public health settings (Kneale et al. 2017; van der Graaf et al. 2017). Conversely, PHLARPs said very little on the application of external peer-reviewed literature to local decision-making. This could relate to perceptions that existing literature is not transferable, indicating a misalignment between academic research priorities and the evidence needs of decision-makers (Cheetham et al. 2022; Kneale et al. 2017; van der Graaf et al. 2017). As such, a lack of engagement with the literature should not be interpreted as a lack of desire for evidence to inform public health decisions. Conversely, studies point to a strong appetite for evidence-informed decision-making within local government (Cheetham et al. 2022; Homer et al. 2022). The minimal attention afforded to evidence use within our interviews also likely reflects the complexity associated with the pathways through which different types of evidence diffuse into public health decisions across levels of local government which are not holistically understood.

Of course, for research to impact public health decisions, it needs to be accessible. Our work echoed previous findings which suggest that although some LAs have infrastructure in place to support access to academic literature, many do not (Cheetham et al. 2022; Kneale et al. 2017). Embedded researchers sought to establish links with research organisations both to facilitate access and to provide a range of more diverse research outputs to their colleagues. These findings highlight the value of open-access publishing and the benefits of providing research evidence in a wide range of formats.

## Conclusions

The intention of this research was not to provide a comprehensive summary of all dimensions associated with research activity and motivation, but rather to offer a preliminary framework on which future work could be built. Further research is needed to explore how the dimensions identified in this study are manifested in a wider variety of contexts and intervention designs as well as through the perceptions of decision-makers firsthand. Indeed, a limitation of this study was the indirect method through which we explored research activity and motivation (i.e., through perceptions of embedded researchers). However, this method was also advantageous, as embedded researchers could provide an insider–outsider perspective of research within local government, thereby offering valuable comparative insight.

We found that research activity in LAs is manifested in a variety of behaviours that overlap with, but are distinct from, research institutions. While identifying overlaps is valuable, it would be inappropriate to expect these research processes to fully align. As such, we suggest that research activity should be conceptualised broadly and, in light of our findings, propose the following expanded version of Fynn et al. (2021)’s definition: research activity refers to activities inclusive of accessing, conducting and contributing to research, using evidence from research, and enhancing opportunities for public involvement in research. However, we also caution against the indiscriminate use of the term “research”, which might not be appropriate in all contexts given how it can be narrowly conceptualised by academics and public health practitioners alike.

We found that terminology can directly affect the motivation of public health staff to engage with research by influencing perceptions around feasibility, value, and capability. As such, to foster trust and local buy-in, this language should be meaningful to decision-makers (e.g., “evidence” or “intelligence” might present more locally appropriate alternatives to “research”) and avoid unconstructive assumptions held by either academia or local government. Communication strategies and partnership approaches should be similarly adaptable to account for variation in research capacity, interest, and skills. Ultimately, research activity in local government should be viewed through a lens of pragmatism which acknowledges the realities of local resource constraints and the political landscape in which public health decisions take place.

## Data Availability

Due to the qualitative nature of this research, participants of this study did not agree for their data to be shared publicly, so supporting data are not available.
